# Detailed Structural Analysis of Lipids Directly on Tissue Specimens Using a MALDI-SpiralTOF-Reflectron TOF Mass Spectrometer

**DOI:** 10.1371/journal.pone.0037107

**Published:** 2012-05-18

**Authors:** Shuichi Shimma, Ayumi Kubo, Takaya Satoh, Michisato Toyoda

**Affiliations:** 1 Venture Business Laboratory, Osaka University, Suita, Osaka, Japan; 2 JEOL Ltd., Akishima, Tokyo, Japan; 3 Project Research Center for Fundamental Sciences, Graduate School of Science, Osaka University, Toyonaka, Osaka, Japan; The George Washington University, United States of America

## Abstract

Direct tissue analysis using a novel tandem time-of-flight (TOF-TOF) mass spectrometer is described. This system consists of a matrix-assisted laser desorption/ionization ion source, a spiral ion trajectory TOF mass spectrometer “SpiralTOF (STOF)”, a collision cell, and an offset parabolic reflectron (RTOF). The features of this system are high precursor ion selectivity due to a 17-m flight path length in STOF and elimination of post-source decay (PSD) ions. The acceleration energy is 20 keV, so that high-energy collision-induced dissociation (HE-CID) is possible. Elimination of PSD ions allows observation of the product ions inherent to the HE-CID process. By using this tandem TOF instrument, the product ion spectrum of lipids provided detailed structural information of fatty acid residues.

## Introduction

In recent years, direct tissue analysis including tissue profiling and imaging mass spectrometry has been at the forefront of research in mass spectrometry.[1]–[5] A wide variety of instrument types including time-of-flight (TOF) mass spectrometers, [6] quadrupole mass filters, ion traps, [7] orbitraps, [8] and Fourier transform mass spectrometers [9], [10] have been described in the literatures. The mass spectrometers are usually coupled to matrix-assisted laser desorption/ionization (MALDI) or to a secondary ion mass spectrometry ionization source. In addition, ambient ionization techniques have recently been applied to the analysis of live tissues. [11], [12].

Furthermore, tandem mass spectrometry (MS/MS or MS^n^) is considered essential for identifying molecules on tissue surfaces. [7], [13] Collision-induced dissociation (CID) is typically used as the dissociation method. There are two types of CID; low-energy CID (LE-CID) and high-energy CID (HE-CID). The two techniques differ primarily by the acceleration energies imparted to the ions. With the exception of tandem magnetic sector mass spectrometers, [14] MALDI-TOF-TOF is the only technique capable of observing HE-CID fragment ions. [15] Since the ions generated by MALDI often undergo post-source decay (PSD), [16], [17] the fragment ions generated by PSD and HE-CID overlap in the product ion spectrum when conventional MALDI-TOF-TOF instruments are used.

Multi-turn TOF (MULTUM) technology is essential for eliminating PSD ions in HE-CID product ion spectra. [18] MULTUM consists of four electrostatic sectors. Most fragment ions formed by metastable decomposition in the MULTUM part of the spectrometer deviate from the normal flight path due to having a different kinetic energy from that of the precursor ion. Therefore, those ions disappear during flight in the MULTUM part and do not reach the detector.

A novel MALDI-TOF-TOF has recently been developed which has a 17-m spiral ion trajectory. [19] This instrument is a MALDI-SpiralTOF(STOF)-Reflectron TOF (RTOF), which is known as JMS-S3000 (JEOL Ltd., Akishima, Tokyo). This instrument uses the multi-turn type ion optical system as the first TOF mass spectrometer (MS1) and an offset parabolic reflectron as the second TOF mass spectrometer (MS2). This system can generate simple HE-CID product ion spectra because:

1. each fragment pathway can be observed as a single peak by selecting the monoisotopic ion of all precursor ions,
2. HE-CID fragment pathways predominate when the PSD ions are eliminated.

Product ion spectra of peptide standards and a synthetic polymer obtained using MALDI-STOF-RTOF have already been reported. [19] We consider that these features will be truly beneficial for direct tissue analysis, because generated ion species are highly complicated. We performed spot analysis of mouse brain sections to confirm the utility of MS/MS using HE-CID directly on the tissue surface (on-tissue HE-CID) in MALDI-SpiralTOF-RTOF. We also used this approach for the first time to provide a detailed structural analysis of lipids that would not be possible using product ion spectra via the LE-CID process. These studies are described in the present paper, together with general perspectives regarding on-tissue HE-CID.

## Results and Discussion

### Mass Spectra from Tissue Surface in the Positive and Negative Ion Detection Mode

Mass spectra obtained directly on the mouse brain tissue surface are shown in Figure S1. Positive ion mode produced peak patterns characteristic of phospholipids and glycolipids with typical mass resolutions of approximately 40,000 (Figure S1A). The mass spectrum obtained in the negative ion detection mode is also shown in Figure S1B. Both spectra show a large number of signal peaks, but because of the characteristics of STOF, each peak was clearly separated and the noise level was quite low. These results demonstrate that even in tissue analysis, the high mass resolution was sufficient to separate monoisotopic ions easily for the subsequent MS/MS experiment.

### Product Ion Spectra in the Positive Ion Detection Mode on the Brain Surface

**Figure 1:**
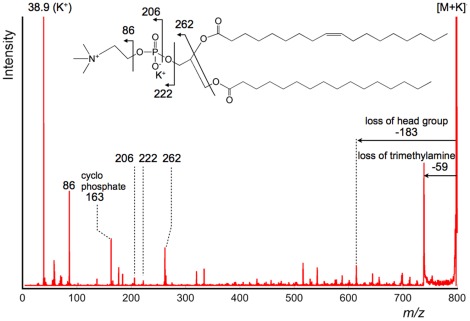
Product ion spectrum of *m/z* 798 obtained directly from the tissue surface (positive ion detection mode). In the obtained product ion spectrum, the fragment ions derived from polar head group were detected. This fragment pattern is identical to the product ion spectra of alkali metal adducted PC obtained via LE-CID, however fragment ions derived from fatty acid moieties were detected above *m/z* 500.

Positive ion detection mode provided product ion peaks generated from the precursor ion at *m/z* 798, arising from the potassium ion adduct of phosphatidylcholine (PC) (16∶0, 18∶1). The PC fragment ion patterns obtained on the tissue surface using LE-CID in ion trap type instruments have been previously reported. [7], [13] The results indicated that the fragment ions derived from the polar head group and neutral loss of fatty acids were predominantly observed in LE-CID. The product ion spectrum of the tissue surface obtained by HE-CID is shown in Figure 1. As shown in the Figure 1 inset, several intense peaks were derived from the polar head group. This peak pattern was characteristic of product ion spectra due to alkali-metal adducts of PC.[20]–[22] In the product ion spectra obtained using TOF-TOF instrument, we could unambiguously discriminate the adduct metal. In Figure 1, the peak at *m/z* 38.9 was derived from potassium. On the other hand in ion trap type instruments, the peaks in the low *m/z* region are below the low mass cut-off. [23] The excellent ion selectivity and wide *m/z* range detection capability in MS/MS were advantages in this system.

**Figure 2:**
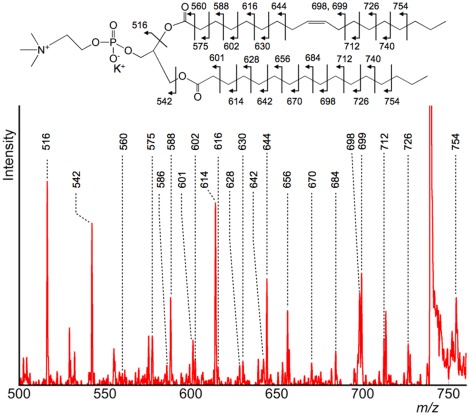
Detailed product ion spectrum of the *m/z* 500–750 region. This product ion spectrum is an enlarged view of the *m/z* 500–750 region in Figure 1. These fragment ions were produced via a charge-remote fragmentation process (HE-CID specific process). The double bond position was recognized by analysing the peak pattern.

Dissociation of the polar head group did not provide distinctive differences between LE-CID and HE-CID, although a regular pattern of peaks with masses above *m/z* 500 was obtained. Details of Figure 1 are presented in Figure 2 and clearly show product ions derived from dissociation of both *sn*-1 and *sn*-2 fatty acids. The product ion spectrum of lyso-PC (LPC(18∶1)) via the HE-CID process has been reported, [24] but our findings are the first report of the HE-CID spectrum of diacyl-PC. To validate this result, we confirmed the pattern of product ion spectra using a standard sample of PC(16∶0, 18∶1). The result is shown in Figure S2. Comparing the data from standard sample and tissue sample, the fragmentation patterns were almost identical. Therefore, the product ion spectra shown in Figure 1 and Figure 2 were derived from potassium adducted PC(16∶0, 18∶1).

The dissociation of fatty acids is known to occur through charge-remote fragmentation (CRF), a characteristic process in HE-CID. In the MALDI-STOF-RTOF system, the ions were accelerated at 20 keV (laboratory frame). Under that condition, the maximum centre-of-mass collision energy (*E_cm_*) between PC(16∶0, 18∶1) and helium gas was calculated as 100 eV. [25] In HE-CID, the internal energy distribution is broader than that of LE-CID. Due to this broad distribution, the probability of various bonds cleavage is increased. Especially, the estimated energy was 12 eV to dissociate a C-C bond. [26] Therefore, the ions had sufficient energy to dissociate fatty acids in this experiment. While in LE-CID, the value of *E_cm_* is generally much lower and the distribution is narrower. The feature in LE-CID is interpreted as occurrence of prior dissociation in the facile bonds like phosphocholine and fatty acid moieties. Consequently, the peaks in the product ion spectra shown in Figure 2 were not produced by LE-CID. [7].

CFR of several fatty acids has been studied using magnetic sector mass spectrometers, [27] and it has been reported by Gross et al. [28] that CRF can be accounted for by a 1,4-H_2_ elimination and by Wysocki and Ross [29] by a homolytic fragmentation mechanism. In PC lipids, the positive charge is fixed on the trimethylamine, allowing both *sn*-1 and *sn*-2 fatty acids to dissociate. Each product ion was assigned as shown in the Figure 2 inset. The full sequence of the fatty acid moiety was included in the product ions via the CRF process, and the position of the double bond in the fatty acid was identified by its resonance-stabilized radical anion (*m/z* 699). [27].

Furthermore, we investigated whether *sn*-1 and *sn*-2 positions for the fatty acid residues could be determined. To answer this issue, we compared product ion spectra obtained from both PC(16∶0, 18∶1) and PC(18∶1, 16∶0) standard samples. The results are shown in Figure S3. According to the report in the LE-CID study, [22] the relative peak intensities derived from the fatty acid moiety (*sn*-1 and *sn*-2) were different. The peak intensity derived from elimination of the fatty acid moiety as a ketene at *sn*-2 (-R^’^_2_CH = C = O) was more abundant compared with the peak intensity from *sn*-1. Our results suggested that this feature was applicable in HE-CID. As shown in Figure S3, the *m/z* values of the corresponding peaks were *m/z* 516.4 and *m/z* 542.4, respectively. The peak intensity derived from elimination at *sn*-2 was always abundant even in HE-CID. These capabilities of determining double bond and *sn*-1 and *sn*-2 positions have reinvigorated interest in the role of lipids in biology. [30], [31].

### Evaluation of Sensitivity for Charge-Remote Fragmentation Using the Standard Analyte

**Figure 3:**
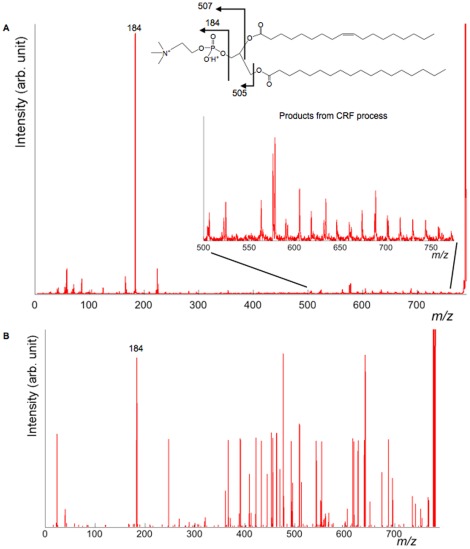
Product ion spectra of PC(18∶0, 18∶1). (A) 10 pmol/µl; (B) 10 fmol/µl. For the sensitivity evaluation in HE-CID, product ion spectra of PC(18∶0, 18∶1) were obtained. In 10 fmol/µl, peak intensities of product ions were below noise level.

In this section, we would like to discuss the sensitivity for product ions derived from the CRF process. As mentioned above, HE-CID could provide characteristic structural information, especially in fatty acid moieties. However, its dissociation efficiency was lower than compared to LE-CID due to short reaction time between the collision gas and the analyte. We considered that this efficiency directly affected the sensitivity. To estimate the sensitivity, we prepared the different concentration standard analytes of PC(18∶0, 18∶1). The concentrations were from 10 fmol/µL, 100 fmol/µL, 1 pmol/µL and 10 pmol/µL. In this experiment, protonated PC(18∶0, 18∶1) ([M+H]^+^788.6) were isolated. Then, product ion spectra were obtained. In the product ion spectra of protonated PC species, the most intense product peak was *m/z* 184 (the base peak of the product ions), which was derived from phosphocholine (Figure 3A). It is difficult to define the sensitivity of the product ion detection. However, we investigated the number of the detected peaks between *m/z* 500–775. As shown in Figure 3A inset, the peaks derived from the acyl chains were detected in this window. Additionally, we investigated relative intensities of total product ions of *m/z* 500–775 and *m/z* 184. When helium gas was introduced into the collision cell, the peak intensity of precursor ion attenuated by 50% compared before and after introduction. Under this condition, the detected number of CRF peaks in 10 fmol/µL, 100 fmol/µL, 1 pmol/µL and 10 pmol/µL were 0, 5, 28 and 43, respectively. In Figure 3B, the product ion spectrum at 10 fmol/µL is presented. In this spectrum, we obtained the product peak at *m/z* 184, however no product peaks from CRF process were detected. The relative intensities (CRF products to *m/z* 184) were 0%, 28%, 36% and 37%, respectively. We found the relative intensities were decreased below 100 fmol/µL. Considering these results, the detection limit of CRF products was speculated approximately 100 fmol/µL.

### Product Ion Spectra in the Negative Ion Detection Mode on the Brain Surface

**Figure 4:**
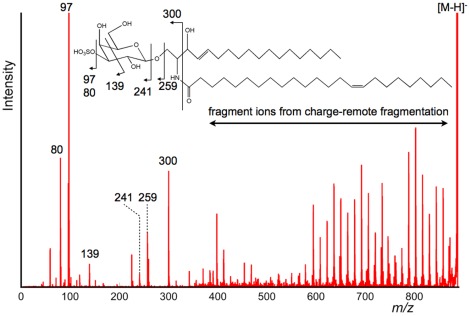
Product ion spectrum of *m/z* 888 obtained directly from the tissue surface (negative ion detection mode). In the obtained product ion spectrum, the fragment ions derived from polar head group were detected. Peaks derived from sulfonic acid and the dissociation products of galactose were observed in the low *m/z* region. Above the region of *m/z* 600, clear unique peak patterns derived from the products of the ceramide moiety were observed.

**Figure 5:**
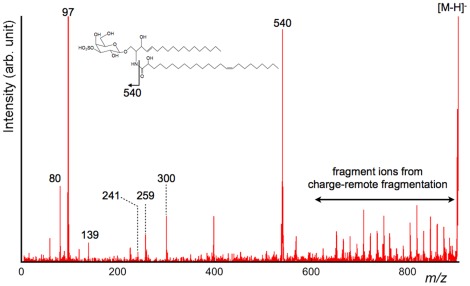
Detailed product ion spectrum of the *m/z* 560–880 region. This product ion spectrum is an enlarged view of the *m/z* 560–880 region in Figure 4. From the peak pattern and the mass value of the precursor ion, *m/z* 888 was postulated as ST(d18∶1, C24∶1). The regular peak patterns obtained via the CRF process were largely assigned as product ions from a fatty acid moiety (C24∶1) and a long-chain base (d18∶1). Furthermore, the carbon–carbon double bond at the *n*−9 position was confirmed from the CRF sequencing pattern.

Having determined that product ions derived from fatty acids could be observed in positive ion detection mode, MS/MS experiments were also performed in negative ion detection mode to determine if product ions could be observed via the CRF process. The product ion spectra of *m/z* 888 and *m/z* 904 obtained directly from the mouse brain surface are depicted in Figure 4 and Figure 5. Since regular peak patterns were clearly observed in both spectra, the results indicated that the dissociation of fatty acids via the CRF process occurred in negative ion detection mode.

The data shown in Figure 4 and Figure 5 suggested that product ions derived from the polar head group would be found in the low *m/z* region. There was a pair of ions of *m/z* 80 and 97, indicating the presence of a sulfate group. Due to the presence of the sulfate ester, the negative charge was considered to be strongly fixed at the position. Therefore, product ions derived from fatty acids via the CRF process were clearly observed in both spectra. Taking into consideration this sulfate group and the regular peak pattern, we concluded that *m/z* 888 and *m/z* 904 were due to a sulfatide (ST), a class of sulfated galactosylceramides. Furthermore, the structure of non-polar part was postulated from the *m/z* value to be (d18∶1, C24∶1) and hydroxy-fatty-acid included species of (d18∶1, C24∶1 h). [32], [33] Interestingly, the peak at *m/z* 540, which corresponded to a lysosulfatide, was clearly observed in Figure 5. This peak was known to as a specific peak in the product ion spectrum of hydroxy-fatty-acid included sulfatides. [7] Even in the HE-CID spectrum of sulfatides, the specific peak was also observed.

**Figure 6:**
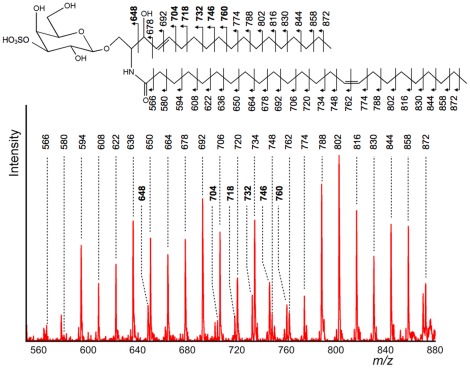
Product ion spectrum of *m/z* 904 obtained directly from the tissue surface (negative ion detection mode). In the obtained product ion spectrum, the fragment ions derived from polar head group were nearly identical to the product ion spectrum of *m/z* 888. Above the region of *m/z* 600, clear unique peak patterns derived from products of the ceramide moiety were also observed. One specific difference between *m/z* 888 and *m/z* 904 was the detection of high-intensity peak at *m/z* 540. This characteristic peak was derived from neutral loss of a hydroxy-fatty-acid.

Based on the assumption of (d18∶1, C24∶1), we attempted to interpret the product ions found between *m/z* 550 and *m/z* 880. The result is shown in Figure 6. Generally, sphingosin (d18∶1) is synthesized from palmitoyl CoA and serine, then oxidized by dihydroceramide desaturase. According to this information, the unsaturated carbons of d18∶1 were determined as shown in Figure 6. On the other hand, C24∶1 could correspond to a nervonic acid, a monounsaturated ω-9 fatty acid. In this case, the unsaturated carbons of C24∶1 would be located at the ninth bond. As expected, most fragment ions could be assigned; in Figure 6, specific peaks in sphingosine are displayed in bold type.

Although most peaks obtained using the CRF process could be assigned, a regular low intensity peaks around *m/z* 800 remained unassigned. A detailed view of this spectral area is shown in Figure S4. The *m/z* values of these unidentified peaks were higher by 2-Da compared to peaks discussed in Figure 6. If the peak interval was one mass unit between neighboring peaks, the peak might be due to products via the homolytic cleavage process. [29] Hence, we postulated that the 2-Da mass shift resulted from different ceramide species with the unsaturated carbon bond in the different positions. According to reports by Colsch et al. [34] and Marsching et al., [35] they suggested that there was a novel sulfatide comprised of shingadienine Δ^4,14^d18∶2 and C24∶0. Especially, Colsch et al. confirmed sphingoid bases composition of sulfatides precisely using precursor ion scans by a triple quadrupole mass spectrometer. Our result might react positively this finding. However, a few unassigned peaks in Figure 6 still remain. There would be other isomeric ceramide species. To study this possibility, we will investigate the fragmentation pattern using synthesized standard samples.

## Materials and Methods

### Chemicals

Methanol was purchased from Wako Pure Chemical Industries (Osaka, Japan). Peptide standards (700–4,000 Da) for external calibration were purchased from Bruker Daltonics (Leipzig, Germany). 3-Aminoquinoline and α-cyano-4-hydroxycinnamic acid (α-CHCA) matrices and the standard sample PC(18∶0, 18∶1) were purchased from Sigma-Aldrich (St. Louis, MO). The standard samples of PC(16∶0, 18∶1) and PC(18∶1, 16∶0) were purchased from Avanti Polar Lipids, Inc (Alabaster, AL). All chemicals used in this study were of highest purity available.

### Matrix Preparation

A liquid matrix reported by Kolli *et al.* was used for MALDI. [36] The matrix solution was prepared by dissolving 35 mg of 3-aminoquinoline in 150 µL methanol saturated with α-CHCA. Matrix solution (0.5 µL) was spotted on the tissue surface.

### Tissue Sample Preparation

**Figure 7:**
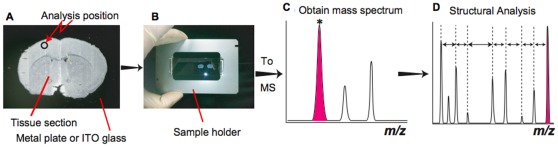
Workflow of direct tissue analysis in this experiment. (A) A frozen tissue section was thaw-mounted on the gold coated metal plate or ITO coated glass plate: (B) the sample plate was fixed on the custom-build sample holder: (C) mass spectrum was obtained in both the positive and negative ion detection mode: (D) HE-CID was performed to obtain structural information of interested ions.

This study used 8-week-old male C57BL/6J mice. The mice were anesthetized with diethyl ether, sacrificed, and dissected. The brain block was immediately frozen in powdered dry ice to minimize degradation and kept at −80°C. Before sectioning, the tissue blocks were left for 30 min at −16°C. Generally, the tissue block was fixed with an optimum cutting temperature (OCT) polymer. However, when the sections were sliced, the cutting block was not embedded in the OCT in this experiment. The tissue sections were sliced in 10 µm-thick sections using a cryostat (CM 3050; Leica, Wetzler, Germany) and thaw-mounted on a gold-coated stainless plate (25 mm×75 mm and thickness of 1.2 mm). The samples were dehydrated in a vacuum chamber and the plate was mounted on a custom-built sample holder. The experimental steps are shown in Figure 7.

### Mass spectrometry

MALDI-STOF-RTOF was equipped with a 349 nm Nd:YLF laser (spot diameter: 20–30 µm) for MALDI, the 17-m-long flight path in STOF for high precursor ion selectivity and PSD ion elimination, and the offset parabolic reflectron including a post-acceleration system to detect product ions. The acceleration voltage in the ion source was 20 kV in both the positive and negative ion detection mode. Precursor ion selection was performed with an ion gate placed 15-m from the ion source. Subsequently, precursor ions entered the collision cell without deceleration, allowing HE-CID. Helium gas was used as the collision gas. A more detailed description of STOF and MALDI-STOF-RTOF has been reported elsewhere. [19], [37], [38].

Mass spectra and product ion spectra were obtained from one spot around the cortex as shown in Figure 7. The data acquisition conditions (i.e., the laser power, collision energy, and number of laser irradiations) were changed as necessary to obtain product ion mass spectra of the fragment peaks that had high intensity and high signal-to-noise ratios (S/N). All mass spectra were externally calibrated, with the external calibration spot deposited on the surface of the metal plate to minimize mass shift.

## Supporting Information

Figure S1 **Mass spectra obtained directly from the mouse brain section.** (A) *m/z* 400–1000 in the positive ion detection mode: (B) *m/z* 700–1000 in the negative ion detection mode. The enlarged spectra of the peaks indicated by asterisks are shown as insets. The typical mass resolution was 40,000. In general, the conventional TOF-TOF instruments have limited precision of precursor ion selection (∼± 3 Da). However, the STOF-RTOF could select only one precursor ion even in the direct tissue analysis.
(TIF)

Figure S2 **Comparison of product ion spectra of** ***m/z*** **798.** (A) Data from PC(16∶0,18∶1) standard sample: (B) data directly from the tissue surface. The fragmentation patterns in both spectra were almost identical.
(TIF)

Figure S3 **Comparison of product ion spectra of isomeric phosphatidylcholine species.** (A) Product ion spectrum obtained from the PC(16∶0, 18∶1) standard: (B) Product ion spectrum obtained from the PC(18∶1, 16∶0) standard. This result indicates that α-hydrogens of the fatty acyl at *sn*-2 are more labile. This feature was reported by Hsu et al. in LE-CID study. Even in HE-CID, this feature is applicable to recognize *sn*-1 and *sn*-2 fatty acids.
(TIF)

Figure S4 **Detailed product ion spectrum of** ***m/z*** **888 in the negative ion detection mode.** Another regular peaks (*m/z* 776, 790, 804, 818 and 832) were observed near main peaks. These peaks had 2-Da higher mass values. Especially, the peak at *m/z* 832 was inferred to be the presence of isomeric ceramide species (d18∶2, C24∶0).
(TIF)
