# Supplementary figures and images for: Detailed Structural Analysis of Lipids Directly on Tissue Specimens Using a MALDI-SpiralTOF-Reflectron TOF Mass Spectrometer
Source: PLoS One. 2012 May 18;7(5):e37107. doi: 10.1371/journal.pone.0037107 (PMC3356408; doi:10.1371/journal.pone.0037107)

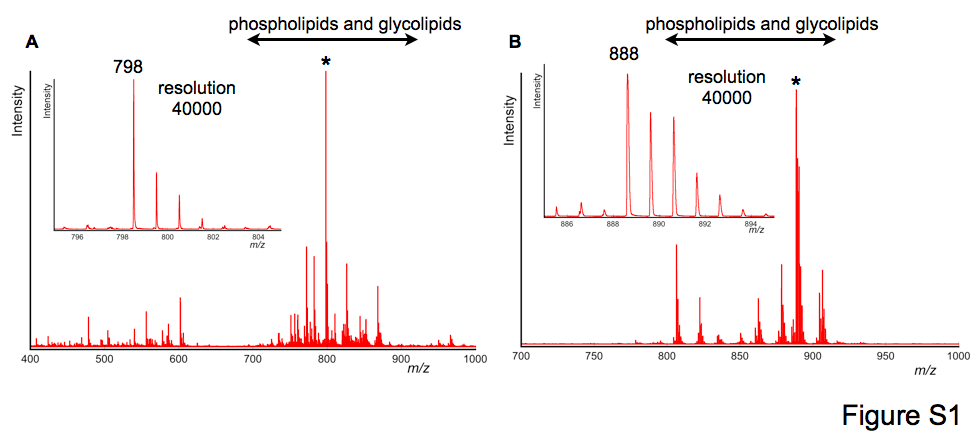

Supplement: Figure S1 — Mass spectra obtained directly from the mouse brain section. (A) m/z 400–1000 in the positive ion detection mode: (B) m/z 700–1000 in the negative ion detection mode. The enlarged spectra of the peaks indicated by asterisks are shown as insets. The typical mass resolution was 40,000. In general, the conventional TOF-TOF instruments have limited precision of precursor ion selection (∼± 3 Da). However, the STOF-RTOF could select only one precursor ion even in the direct tissue analysis. (TIF) [file pone.0037107.s001.tif]

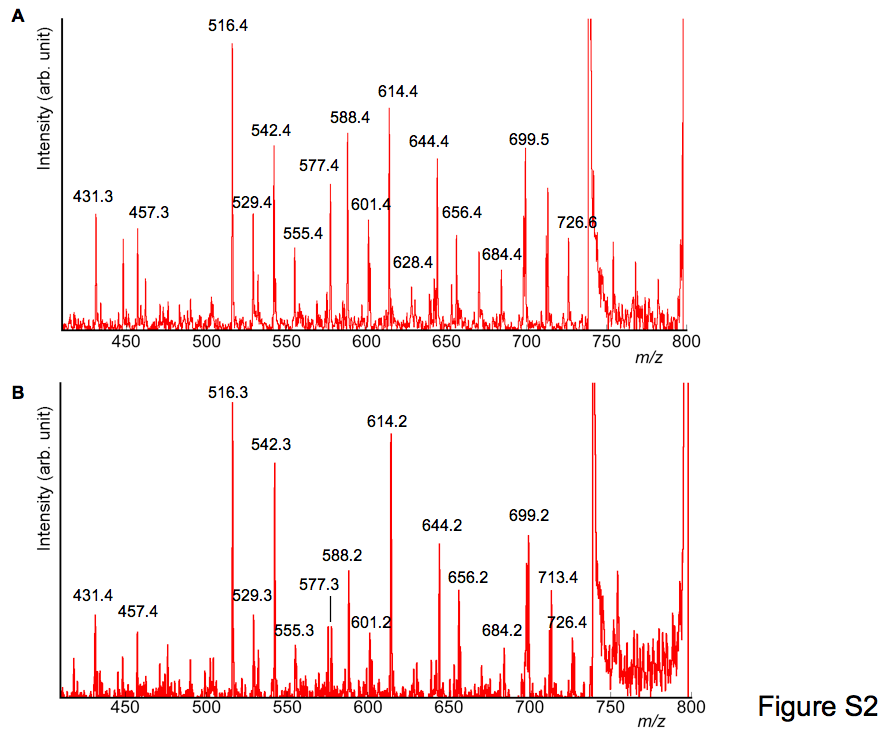

Supplement: Figure S2 — Comparison of product ion spectra of m/z 798. (A) Data from PC(16∶0,18∶1) standard sample: (B) data directly from the tissue surface. The fragmentation patterns in both spectra were almost identical. (TIF) [file pone.0037107.s002.tif]

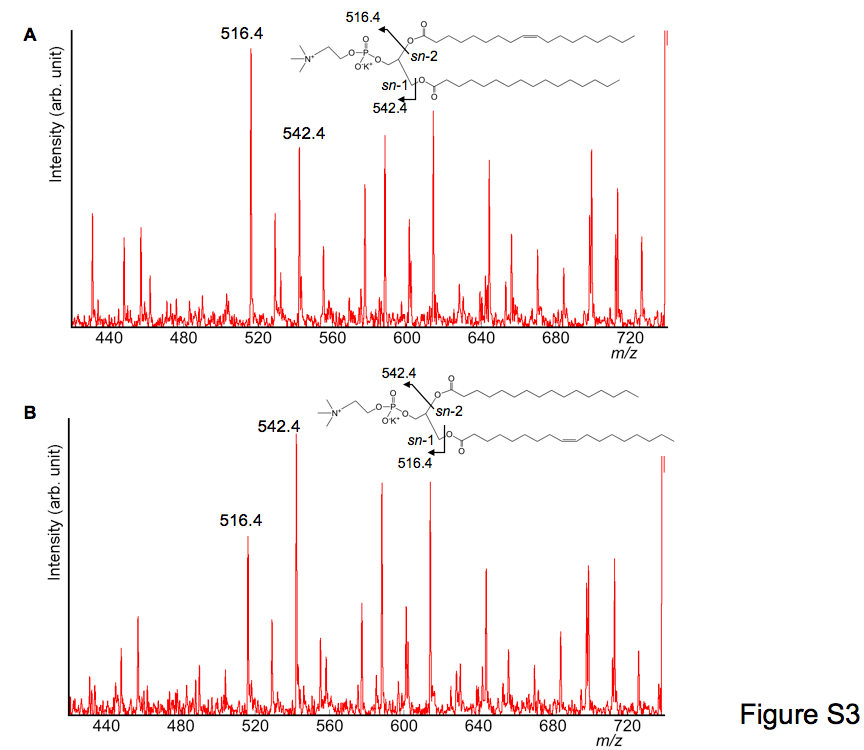

Supplement: Figure S3 — Comparison of product ion spectra of isomeric phosphatidylcholine species. (A) Product ion spectrum obtained from the PC(16∶0, 18∶1) standard: (B) Product ion spectrum obtained from the PC(18∶1, 16∶0) standard. This result indicates that α-hydrogens of the fatty acyl at sn-2 are more labile. This feature was reported by Hsu et al. in LE-CID study. Even in HE-CID, this feature is applicable to recognize sn-1 and sn-2 fatty acids. (TIF) [file pone.0037107.s003.tif]

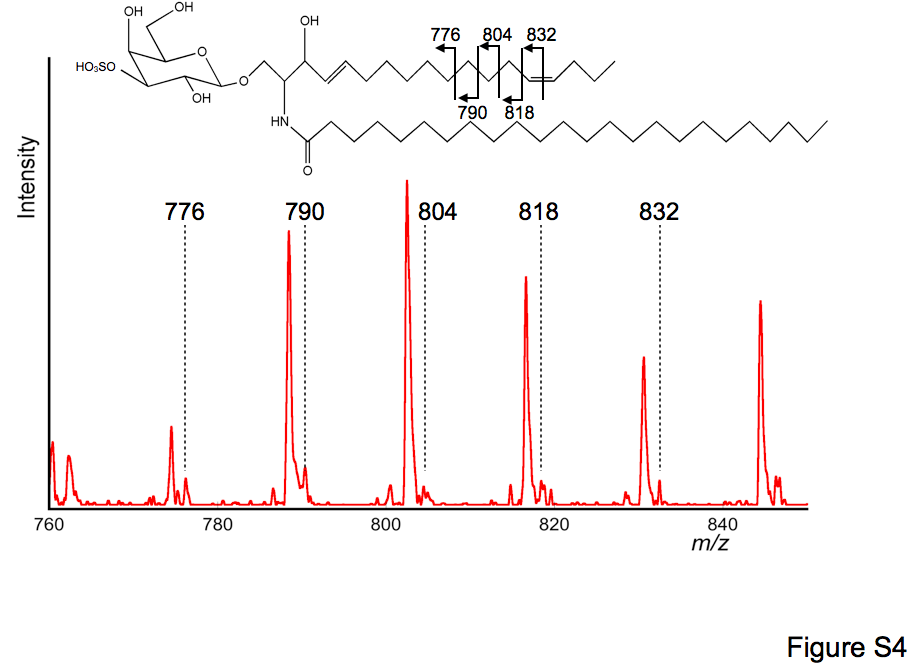

Supplement: Figure S4 — Detailed product ion spectrum of m/z 888 in the negative ion detection mode. Another regular peaks (m/z 776, 790, 804, 818 and 832) were observed near main peaks. These peaks had 2-Da higher mass values. Especially, the peak at m/z 832 was inferred to be the presence of isomeric ceramide species (d18∶2, C24∶0). (TIF) [file pone.0037107.s004.tif]
